# Magnetic targeting as a strategy to enhance therapeutic effects of mesenchymal stromal cells

**DOI:** 10.1186/s13287-017-0523-4

**Published:** 2017-03-09

**Authors:** Luisa H. A. Silva, Fernanda F. Cruz, Marcelo M. Morales, Daniel J. Weiss, Patricia R. M. Rocco

**Affiliations:** 1 Laboratory of Pulmonary Investigation, Carlos Chagas Filho Institute of Biophysics, Federal University of Rio de Janeiro, Av. Carlos Chagas Filho, 373, Ilha do Fundão, Rio de Janeiro, RJ 21941-902 Brazil; 2 Laboratory of Cellular and Molecular Physiology, Carlos Chagas Filho Institute of Biophysics, Federal University of Rio de Janeiro, Av. Carlos Chagas Filho, 373, Ilha do Fundão, Rio de Janeiro, RJ 21941-902 Brazil; 3 Department of Medicine, Vermont Lung Center, College of Medicine, University of Vermont, 89 Beaumont Ave. Given, Burlington, VT 05405 USA

**Keywords:** Mesenchymal stromal cells, Superparamagnetic nanoparticles, Biocompatibility, Magnetic devices, Magnetic targeting, Cell therapy

## Abstract

Mesenchymal stromal cells (MSCs) have been extensively investigated in the field of regenerative medicine. It is known that the success of MSC-based therapies depends primarily on effective cell delivery to the target site where they will secrete vesicles and soluble factors with immunomodulatory and potentially reparative properties. However, some lesions are located in sites that are difficult to access, such as the heart, spinal cord, and joints. Additionally, low MSC retention at target sites makes cell therapy short-lasting and, therefore, less effective. In this context, the magnetic targeting technique has emerged as a new strategy to aid delivery, increase retention, and enhance the effects of MSCs. This approach uses magnetic nanoparticles to magnetize MSCs and static magnetic fields to guide them in vivo, thus promoting more focused, effective, and lasting retention of MSCs at the target site. In the present review, we discuss the magnetic targeting technique, its principles, and the materials most commonly used; we also discuss its potential for MSC enhancement, and safety concerns that should be addressed before it can be applied in clinical practice.

## Background

Mesenchymal stromal cells (MSCs) obtained from various sources, including bone marrow, adipose tissue, placental tissue, and others, have been widely investigated in regenerative medicine research [1]. Besides their ability to differentiate into various mesodermal cell lineages, MSCs also have immunomodulatory properties, acting on innate and adaptive immune cells with resulting attenuation of the inflammatory response [1, 2] which broadens their potential clinical applications.

These immunomodulatory effects of MSCs are predominantly mediated by secretion of paracrine factors that have anti-inflammatory, anti-apoptotic, antifibrotic, and angiogenic properties [1]. These factors include serum proteins, growth factors, hormones, cytokines, extracellular matrix proteases, lipid mediators, messenger RNAs, and microRNAs [3]. Additionally, some of these factors may be secreted into extracellular vesicles—cytosolic fragments with spheroid morphology enclosed by a lipid bilayer [3].

Despite the functionality of MSCs, their therapeutic efficacy in experimental models [4] has not been observed in human patients [5]. These results can be explained by two reasons. First, MSCs are not properly activated in human microenvironments and thus fail to exert immunomodulation and secrete repairing factors. Several strategies have been tested to increase MSC potency, such as cultivation of MSCs in hypoxic conditions [6], in the presence of interferon (IFN)-γ [7], or with serum extracted from patients with respiratory distress syndrome (ARDS) [8]. The second reason concerns the level of difficulty associated with delivery of MSCs and their engraftment at certain target sites, i.e., few MSCs reach the myocardium, spinal cord, and joints after systemic administration, which is a preferred noninvasive route [9]. However, when MSCs were locally administered, long-lasting retention in sites of injury did not occur [10, 11]. It is known that MSCs are dragged through the bloodstream when administered directly to the heart [11]. Since few cells arrive and engraft at the injury site, fewer repairing factors are secreted, thus slowing recovery.

To optimize MSC delivery and retention, the magnetic targeting (MT) technique has been tested. Initially developed to optimize chemotherapeutic procedures, this technique is based on prior magnetization of MSCs followed by in vivo targeting with the aid of magnetic fields. MT would thus enable a larger portion of inoculated cells to reach the site of injury, providing greater and longer lasting release of mediators without the need to increase the cell volume administered [11].

Based on the foregoing, this review aims to discuss the MT technique, explaining its principles and the materials commonly used; we also discuss the potential of MT for enhancement of MSC properties, and safety concerns that should be addressed before MT can be applied in clinical practice.

## The magnetic targeting technique

**Fig. 1:**
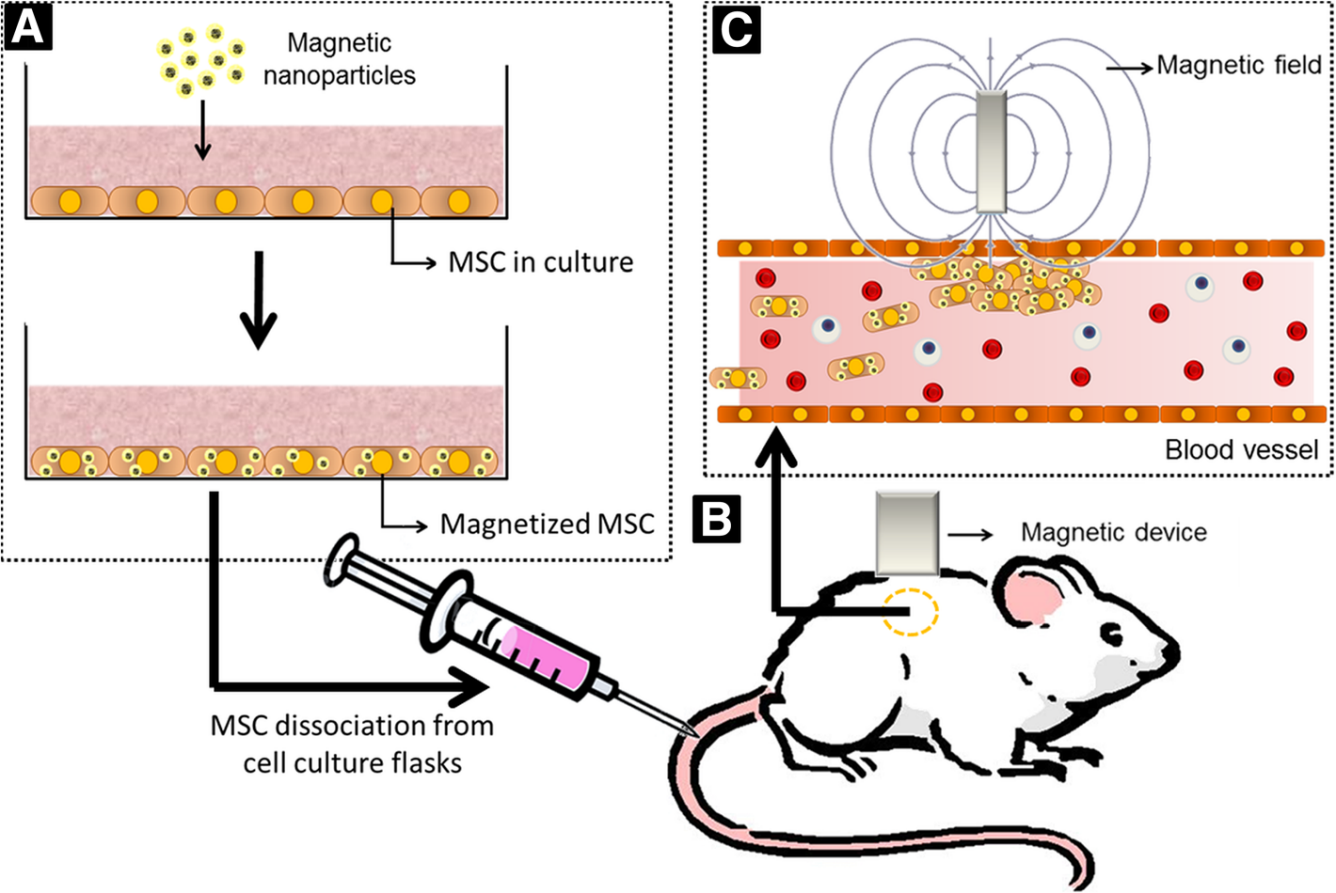
Schematic diagram of magnetic targeting of mesenchymal stromal cells. **a** In the first step, mesenchymal stromal cells (*MSCs*) are expanded in culture and magnetized with magnetic nanoparticles. **b** Once magnetized, the cells are injected into animals which are exposed to static magnetic fields generated by magnetic devices. **c** Magnetized MSCs are better retained in regions where the static magnetic field is present

Briefly, MT of MSCs involves three steps: 1) isolation, growth, and maintenance of MSCs in culture; 2) magnetization of MSCs; and 3) in vivo guidance of magnetized MSCs by static magnetic fields [12]. A schematic diagram is provided in Fig. 1.

### MSC magnetization with superparamagnetic nanoparticles

Magnetization is achieved by diluting magnetic nanoparticles (MNPs) in cell culture medium and cocultivating them with MSCs. The MSCs internalize the nanoparticles by passive diffusion or endocytosis, generally over a few hours [13]. Some of the materials commonly used for the production of these MNPs—iron, nickel, and cobalt—may be toxic to the cells themselves or for in vivo application [14]. However, among these, the iron oxides magnetite (Fe_3_O_4_) and maghemite (γ-Fe_2_O_3_) are considered safer components [15]. Furthermore, maghemite MNPs cause less damage to receptor cells compared to magnetite by having iron in an oxidized state (Fe^3+^) [16].

MNPs smaller than 30 nm are superparamagnetic, i.e., their magnetization only occurs in the presence of an external magnetic field [16]. This is a desirable property for biological applications. Thus, these small superparamagnetic nanoparticles (SPIONs) are essential materials for potential clinical applications of MT-augmented MSC-based cell therapies. In addition, they can also be used as contrast agents for MSC labeling and tracking in vivo due to the strong signal they generate in magnetic resonance imaging (MRI) [16].

Importantly, SPION aggregation in cell culture media can hinder MSC magnetization since their availability for contact with the cells decreases; furthermore, when SPIONs agglomerate, the size of the resulting cluster is similar to or larger than MSCs, thus resulting in a physical restriction for MSC uptake [17]. To minimize such clumping and to promote solubility in aqueous media and in physiological conditions, SPIONs are coated with biocompatible substances (dextran, carboxidextran, polyethylene glycol, polystyrene, silica, etc.) [15]. Importantly, the charge of the SPION coating agent influences their uptake by MSCs: cationic agents interact electrostatically with the negative cell membrane and promote adsorptive endocytosis. In contrast, dextran-coated SPIONs, which are neutral, are not well captured [18]. However, there are additional strategies to optimize the uptake of neutral SPIONs which include physical (electroporation, microinjection, and magnetofection) and biochemical methods (conjugation of antibodies, peptides, or aptamers) [18].

To date, eight different MNPs have been tested for MT of MSCs (Table 1). Most of these are commercially available and/or US Food and Drug Administration (FDA) approved [12]. The commercially available SPION ferucarbotran (Resovist®, Bayer Schering Pharma AG), a carboxydextran-coated SPION with crystalline diameter ranging from 45 to 60 nm, has been most investigated.

**Table 1:** Application of magnetic targeting techniques in pre-clinical studies

Target	MSC donor	MSC recipient	Nanoparticle	Magnetic device	Magnetic device position	Reference
Knee joint (cartilage)	Human	Pigs and rabbits	Feridex (Tanabe Seiyaku)	Electromagnet	External	[10]
	Rabbit	Rabbits	Ferucarbotran/Resovist®	Permanent magnet	External	[24]
Meniscus	Rabbit	Rabbit	Ferucarbotran/Resovist®	Permanent magnet	External	[25]
Skeletal muscle	Human	Rats	Risovist®	Electromagnet	External	[23]
Bones	Rat	Rats	Ferucarbotran/Resovist®	Permanent magnet	External	[26]
Spinal cord	Rat	Rats	Poly-l-lysine-coated SPIONs	Permanent magnet	Implanted	[21]
	Not reported	Rats	Not reported	Permanent magnet	External	[20]
Retina	Rat	Rats	FluidMAG-D®	Permanent magnet	Implanted	[27]
Arteries	Rabbit	Rabbits	FluidMAG-D®	Permanent magnet	External	[29]
Heart	Rat	Rats	Ferucarbotran/Resovist®	Permanent magnet	External	[49]
	Pig	Pigs	Gadolinium nanotubes and Molday ION(–)®	Permanent magnet	Implanted	[22]
	Rat	Rats	Ferucarbotran/Resovist®	Permanent magnet	External	[11]
Lung	Human	Mouse	DMSA-coated maghemite nanoparticles	Permanent magnet	External	[33]

*DMSA* dimercaptosuccinic acid, *MSC* mesenchymal stromal cell, *SPION* superparamagnetic iron oxide nanoparticle

### Static magnetic fields

Once magnetized, MSCs can be guided in vivo by being attracted by external static magnetic fields (SMFs). The intensity of these SMFs is categorized, according to their power induction, as weak (<0.001 Tesla (T)), moderate (0.001–1 T), strong (1–5 T), and ultra-strong (>5 T). Moderate SMFs are those most exploited for clinical MT purposes [19].

Two types of magnetic devices have been used to generate SMFs for MT of MSCs: permanent magnets and electromagnets [12]. Permanent magnets are materials that produce a persistent magnetic field independently of any external magnetic fields. In the context of MT, permanent rare-earth magnets (neodymium-iron-boron) are usually used to generate SMFs since they are portable, reach higher field strengths compared to electromagnets of similar size, and do not require a power supply or cooling system [12]. These magnets can be placed externally above the target site [20] or internally under the skin [21, 22]. However, most studies opt to place magnets externally to avoid the risk of implantation surgery [12, 22] (Table 1). It is also important to mention that the SMFs provided by permanent magnets decay over distance and, therefore, do not reach the inner most parts of the body. To address this issue, placement of multiple permanent magnets in different positions to extend SMF reach has been used, enabling cell delivery to regions such as the spinal cord [20].

Unlike a permanent magnet, an electromagnet only exhibits magnetism when an electric current is flowing through it. SMFs generated by electromagnets have been widely used for MT therapy targeting muscles and joints, being placed externally on the target region, promoting focused MSC retention [10, 23]. Moreover, electromagnets can produce much higher field strengths than permanent magnets; however, they require a constant power supply and must be supercooled to maintain low resistance and prevent overheating [12].

Computational simulations have shown the potential of MRI systems to magnetically guide stem cells [12], an interesting possibility that would allow magnetically targeted cells to home to specific portions of the body while simultaneously providing information regarding in vivo MSC localization. Therefore, ideally, a SPION should be chosen that provides localization information on MRI while making receptor MSCs magnetically responsive.

## Benefits of magnetic targeting in experimental studies

MT of MSCs has been trialed most often for the repair of articular cartilages which have limited healing potential [10, 24] (Table 1). Intra-articular MSC injections into cartilage defect regions resulted in poor engraftment, suggesting the need for inoculation of additional cell volumes. However, administration of larger MSC amounts can generate loose bodies of fibrotic tissue, affecting joint biomechanics [10]. Therefore, for safe treatment, appropriate numbers of MSCs must be transplanted efficiently into the joint.

SMFs from extracorporeal permanent magnets placed next to the knee joint were found to provide better engraftment of SPION-labeled MSCs after their injection into cartilage degeneration sites. Additionally, MT avoids MSC migration to any major organs and formation of loose bodies [10, 24].

Likewise, MT with external permanent magnets/electromagnets has been shown to enhance MSC engraftment in other parts of the musculoskeletal system, such as fibrocartilages (meniscus) [25], muscles [23], and bones [26] (Table 1). These areas also have limited regenerative capacity and may be poorly accessible by systemic or local MSC inoculation.

MT has also been tested in models of central nervous system lesions. In rat models of spinal cord injury, SPION-labeled MSCs were administered intrathecally and guided by implanted [21] or external [20] permanent magnets to the injured sites (Table 1). In one of these studies, histological analysis showed significantly higher MSC counts at the lesion site with the aid of implanted magnets (9595 ± 2231 cells) than in control groups (3538 ± 625 cells) 12 h after cell administration [21]. Importantly, MSC retention was uniform and concentrated in regions close to the injury sites [20, 21]. However, whether this enhancement led to clinical improvement was not assessed.

In one study, intravenously administered MSCs were successfully guided into small dystrophic areas of the retina with the aid of implanted magnets; MSC counts over the retinal surface were 10-fold greater than in animals without magnets [27]. Therefore, MT of MSCs resulted in higher retinal concentrations of anti-inflammatory molecules, such as interleukin-10 and hepatocyte growth factor, providing evidence of a significant therapeutic benefit in the dystrophic retina model [27].

In cardiovascular diseases, MSCs retention is less than 10% after 24 h and long-term engraftment is even more infrequent since these cells are usually delivered by the intravascular route and, thus, are subject to a washout effect caused by heart contractions and venous drainage [28]. Nevertheless, MT may improve cardiac retention of MSCs through cell magnetization and placement of magnets either on the heart or injured vessels [28] (Table 1).

The potential of MSC MT to reduce the risk of restenosis and reocclusion of treated vessels after angioplasty has been assessed [29]. In a femoral artery injury model, permanent magnets were placed externally on the leg at the site of injury and remained in place for 24 h while cells were injected directly into the diseased artery. This technique led to a sixfold increase in MSC retention, avoiding the washout effect, and a reduction in restenosis 3 weeks after cell injection [29].

Magnetically targeted MSCs were also found to provide enhanced therapeutic benefit in models of myocardial infarction [11, 22]. In one report, transplanted MSC counts in the ventricular wall were approximately 3.04-times greater than those measured in control groups (25.8 ± 4.7 versus 8.5 ± 2.0). As a consequence, left ventricular remodeling was attenuated and cardiac function was ameliorated [11]. In these studies, the cells were injected intravenously or locally into the epicardium, while permanent magnets were placed internally, close to the target region, without impairing cardiac function [11, 22].

Some mechanisms may explain why MT potentiated the therapeutic effects of MSCs in these studies. First, MT enhances MSC retention in injury sites as a consequence of magnetic interactions between magnetized cells and SMFs and MSC gene expression changes [13]. One hour after SMF exposure, in vitro, magnetized MSCs presented increased expression of integrins (alpha 2, alpha 6, and beta 3), adhesion molecules (intercellular adhesion molecule-2 and platelet endothelial cell adhesion molecule), and other proteins, such as CD93 (involved in innate immunity, inflammation, and adhesion to endothelium) and cadherin 7 (involved in cell adhesion, dispersion, and migration) [30, 31]. These changes may contribute to increased adherence and engraftment to target sites in vivo, which is particularly interesting for cardiac and orthopedic applications. Therefore, higher MSC retention would arguably result in greater release of soluble factors and restorative action. Secondly, SMFs increase secretion of membrane-derived extracellular vesicles by MSCs in vitro, as well as induce changes in their content [32]. The vesicles derived from MSCs exposed to SMFs are richer in some specific growth factors, including bone morphogenetic protein 2 (BMP-2) and vascular endothelial growth factor (VEGF) [32]. These factors may all play therapeutic roles in cardiovascular and musculoskeletal disorders.

Although magnetically targeted MSCs have shown positive effects in vivo, the MT technique has yet to be extensively investigated in other experimental models, such as lung and liver diseases (Table 1). In a recent report, MT was found to enhance MSC retention in murine lungs [33]. For MT, permanent magnets were attached to the dorsal region, above the thorax, and SPION-labeled MSCs were immediately administered via the jugular vein [33]. The findings of this experiment suggest that MT has potential to guide MSCs to injured areas in lungs.

There are no reports on the use of MT in cell therapy for renal diseases. This may be explained by the ease of access of MSCs to the kidneys after systemic administration due to capillary trapping, which eliminates the need for techniques to increase engraftment [9].

## Concerns

Even though MT has been shown to potentiate the therapeutic effects of MSCs in different experimental models, some safety concerns need to be addressed prior to conducting clinical trials with this technique. These include issues of biocompatibility between MSCs and SPIONs, the influence of SMFs on MSCs, and in vivo adverse effects.

### SPION-MSC interactions

When excess ferric or ferrous ions accumulate in the cytoplasm in noncomplexed form they catalyze biomolecular oxidation reactions, increasing the rate of free-radical generation [34]. These radicals can irreversibly modify amino acids, denature or aggregate proteins, oxidize nucleotides, and promote lipid peroxidation [34]. Nevertheless, none of the SPIONs used in MSC MT studies have been shown to exert toxic effects on recipient cells [33, 35–37].

SPIONs are biocompatible with MSCs due to their surface chemical modifications. Capping agents are used not only to ensure SPION stability in physiological media, but also to keep the iron core isolated from biomolecules, enhancing the safety of this material for biological applications [38]. SPION toxicity, therefore, is dependent on coating agent stability in culture medium or after cell uptake; if the nanoparticle coating is easily degraded, the metal core is then free to react with biomolecules. Importantly, cationic or anionic capping agents, such as dimercaptosuccinic acid (DMSA), are more difficult to remove from the nanostructure compared to neutral substances, such as dextran or albumin [38].

Another reason for SPION biocompatibility is that these nanoparticles may have an activity similar to that of natural catalases, which, importantly, depends on the acidity of the surrounding cellular environment [39].

Looking beyond toxicological issues, the potential impacts of SPIONs on the fundamental biological features of MSCs, such as proliferation, immunomodulation, and differentiation, are less well understood. Ferucarbotran has been shown to stimulate in vitro MSC proliferation [40], exert an inhibitory effect on osteogenesis [41] and chondrogenesis [42], and to reduce cell migration potential and colony-forming ability [35]. It is important to note that these studies used different SPION concentrations and times of exposure, thus precluding comparison of dose-response and time-response patterns.

Furthermore, infusion of MSCs labeled with a SPION for MRI tracking purposes in a rat model of multiple sclerosis led to aggravation of symptoms, whereas unlabeled MSCs ameliorated symptoms [43]. One hypothesis is that SPIONs increased free-radical release, intensifying inflammatory responses and accelerating disease progression [43]. Given these observations, it is important to carry out biocompatibility tests prior to in vivo experiments and clinical trials so as to mitigate adverse effects related to SPION-MSC interactions.

### Effects of SMFs on MSC viability and function

Recently, several studies have been conducted to assess how SMFs influence biological systems [44]. It was found that SMFs can affect the rotation of cell membrane phospholipids by virtue of their diamagnetic properties. This leads to changes in cell shape, cytoskeletal rearrangement, and alterations in ion channel function. Through these ion channel changes, SMFs can decrease intracellular calcium ion concentrations, which may explain some of their observed effects, including modulation of apoptosis, proliferation, and cell viability [45]. Importantly, the type and extent of modifications in cell shape and function depends on cell type and age, field strength, and time of exposure [44].

**Fig. 2:**
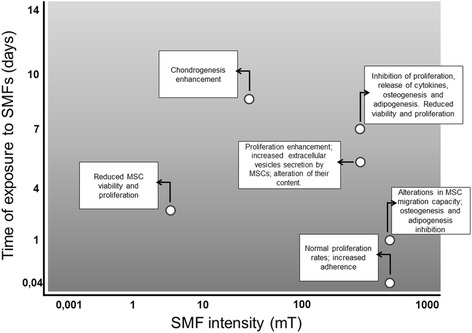
Effects of static magnetic fields (*SMFs*) on mesenchymal stromal cells (*MSCs*). The combination of different field intensities (*x*-axis) and different exposure times (*y*-axis) promotes varying effects on these cells

These facts raise concerns about the potential influences of SMFs on the biological functions of MSCs and on patients as a whole. To date, reports of SMF use with MSCs have demonstrated that moderate SMFs can have divergent effects (enhancing or inhibiting) on MSC viability [19, 45], proliferation [45–47], differentiation capacity [19, 31, 47, 48], colony formation [31], and extracellular vesicle secretion [19, 46] (summarized in Table 2 and Fig. 2). In addition, the risk of vascular embolisms must be considered [49]. In an ischemic rat model subjected to intracavitary SPION-labeled MSC injection, high SMF intensities induced cell accumulation in the vessel lumen [49, 50]. In this study, a permanent magnet was placed next to the injured myocardium for only 20 min (10 min after and 10 min before cell injection).

**Table 2:** Effects of SPIONs and static magnetic fields on mesenchymal stromal cell properties

MSC origin	Nanoparticle	Magnetic device	SMF strength (mT)	Time of exposure	Effects of SMFs on MSC (compared to control groups)	Reference
Human bone marrow	Ferucarbotran/Resovist® (60 μg/ml)	Permanent magnet	600	24 hours and 12 days	Reduction of colony-forming units, increased adipogenesis, and osteogenesis inhibition	[31]
Human bone marrow	Feridex (Tanabe Seiyaku)	Electromagnet	600	1 hour	Increased expression of integrins and adhesion proteins	[30]
Murine bone marrow	None	Electromagnet	4, 7, and 15	1 to 4 days	Reduction of MSC viability and proliferation rates	[45]
Canine and equine adipose tissue	None	Permanent magnet	500	1 to 7 days	Increased MSC proliferation rates in both species; increased secretion of extracellular vesicles by equine MSCs	[46]
Human bone marrow	None	Permanent magnet	400	14 days	Increased chondrogenesis	[48]
Equine adipose tissue	None	Permanent magnet	500	1 to 7 days	Ultrastructural changes; increased proliferation rate, colony-forming units, and secretion of extracellular vesicles; changes in vesicle content.	[32]
Human bone marrow	None	Permanent magnet	3, 15, and 50	1 to 9 days	Increased MSC proliferation rates; osteogenesis stimulation.	[47]
Murine adipose tissue	Feridex (Berlex)	Permanent magnet	500	7 days	Reduction of MSC viability, proliferation rates, angiogenic cytokine release, osteogenesis and adipogenesis; phenotype shift.	[18]

*MSC* mesenchymal stromal cell, *SMF* static magnetic field, *SPION* superparamagnetic iron oxide nanoparticle

Finally, it must be noted that these studies used MSCs isolated from different tissues, were conducted in various species, and applied different SMF intensities during various exposure times. Thus, comparisons are extremely limited. Nevertheless, the adverse effects of SMFs on MSCs can be mitigated by reducing exposure time [28]. For example, MSCs labeled with SPIONs and subjected to a moderate SMF (0.6 T) for 1 h did not exhibit changes in proliferation rate over 3 weeks, indicating viability [30]. Any potential complications of SMF and SPION effects on MSCs in vitro and in vivo must be thoroughly investigated and overcome before clinical use.

## Conclusions

The intensity and duration of the beneficial effects of clinical MSC administration may be enhanced by MT, due to the efficacy of this technique in guiding cells to disease foci, improving their retention and engraftment, and, possibly, enhancing immunomodulatory properties. Therefore, MT is a potentially exciting approach for improving the efficacy of MSC-based cell therapies. However, there is still much to learn about the optimal use of MT with MSCs and minimizing or eliminating any potential adverse effects.

## Abbreviations

MNP: Magnetic nanoparticle
MRI: Magnetic resonance imaging
MSC: Mesenchymal stromal cell
MT: Magnetic targeting
SMF: Static magnetic field
SPION: Superparamagnetic iron oxide nanoparticle
